# Supplementary material for: Investigation of Stabilized Amorphous Solid Dispersions to Improve Oral Olaparib Absorption
Source: Pharmaceutics. 2024 Jul 19;16(7):958. doi: 10.3390/pharmaceutics16070958 (PMC11280475; doi:10.3390/pharmaceutics16070958)
Supplement: Supplementary file 1 [file pharmaceutics-16-00958-s001.zip › pharmaceutics-3061329-supplementary.pdf]

**Supplementary information**

# Investigation of Stabilized Amorphous Solid Dispersions to Improve Oral Olaparib Absorption

**Taehan Yun, Sumin Lee, Seowan Yun, Daeyeong Cho, Kyuho Bang * and Kyeongsoo Kim ***

Department of Pharmaceutical Engineering, Gyeongsang National University, 33 Dongjin-ro, Jinju 52725, Republic of Korea

* Correspondence: khbang0095@gnu.ac.kr (K.B.); soyoyu79@gnu.ac.kr (K.K.)

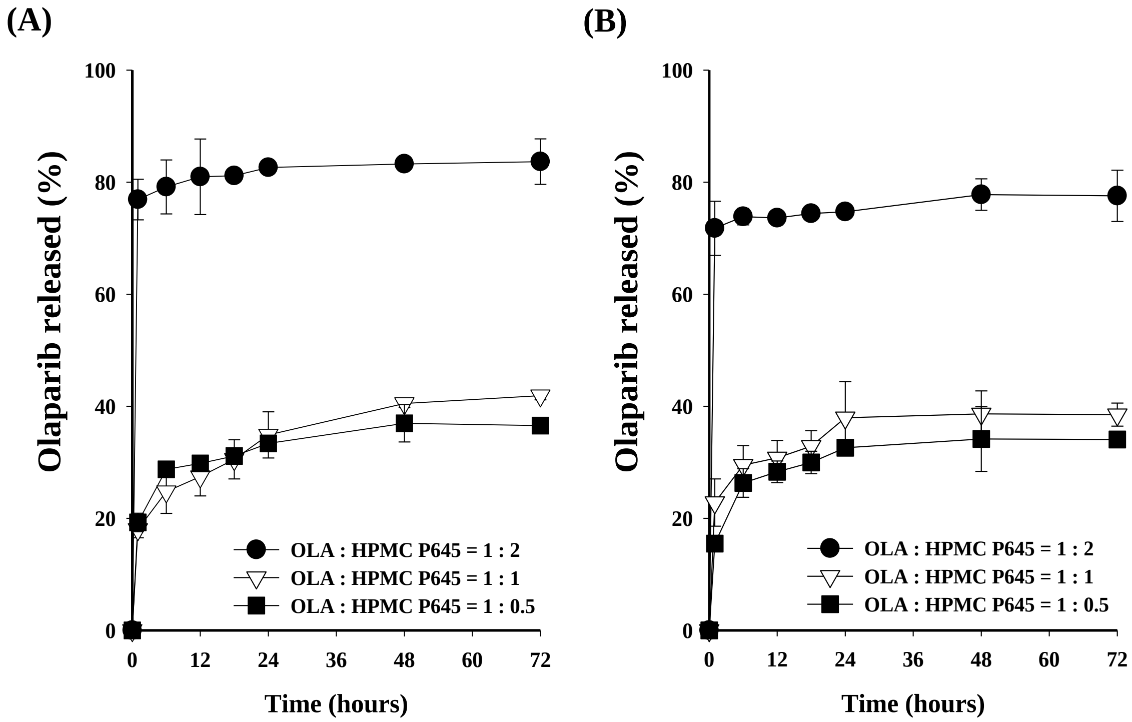


**Figure S1.** The dissolution profile from HPMC P645 at different ratios under (A) pH 1.2 and (B) pH 6.8.

**(A)**

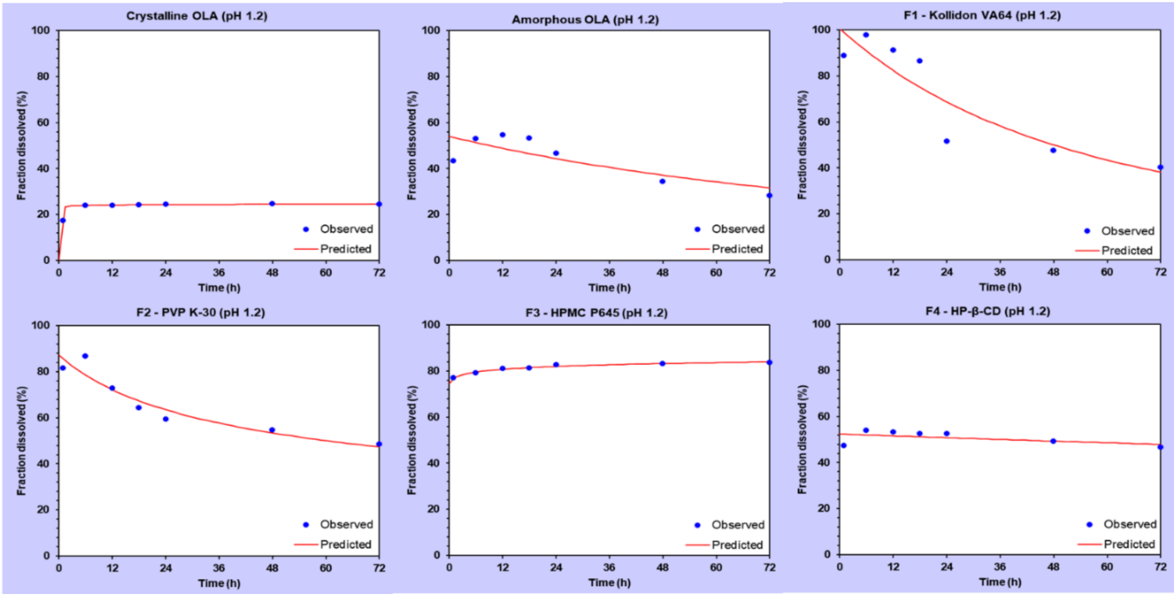


**(B)**

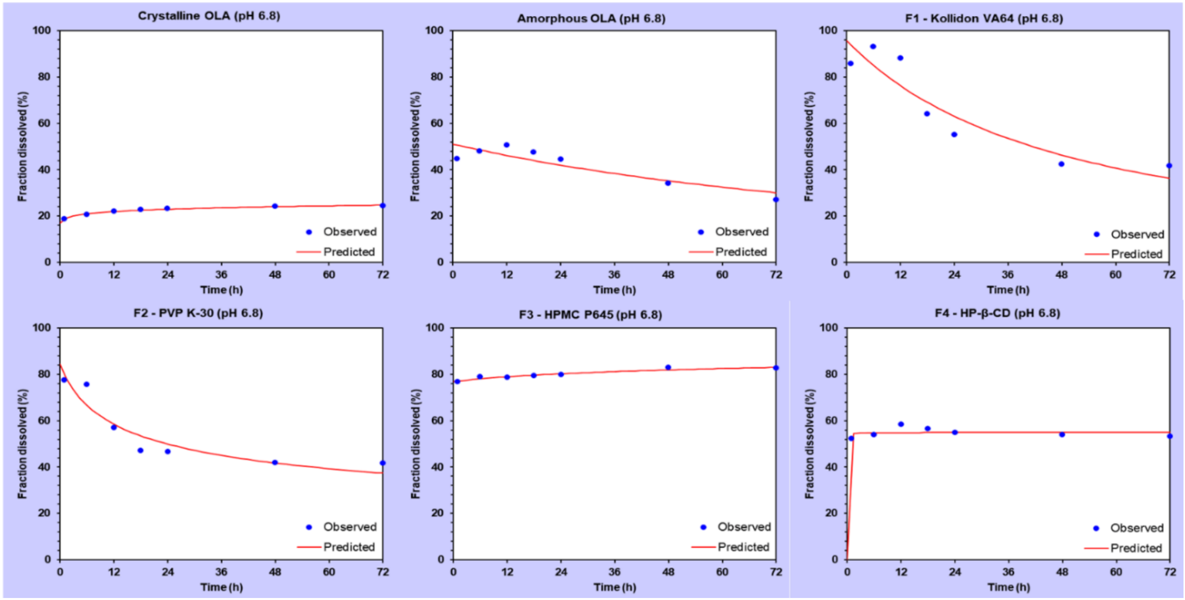


**Figure S2.** Korsmeyer–Peppas model with Talg release kinetics of OLA-SD formulations at (A) pH 1.2 and (B) pH 6.8.

**Table S1.** R-squared values of the Korsmeyer–Peppas model with Tlag for each sample.

| Formulation | $R^2$ (pH 1.2) | $R^2$ (pH 6.8) |
|---|---|---|
| Crystalline OLA | 0.998 | 0.984 |
| Amorphous OLA | 0.665 | 0.804 |
| F1 (Kollidon VA64) | 0.811 | 0.861 |
| F2 (PVP K-30) | 0.905 | 0.888 |
| F3 (HPMC P645) | 0.977 | 0.924 |
| F4 (HP-β-CD) | 0.302 | 0.226 |
